# Green Supply Chain Optimization Based on BP Neural Network

**DOI:** 10.3389/fnbot.2022.865693

**Published:** 2022-05-30

**Authors:** Huan Wang

**Affiliations:** College of Economics and Management, Hubei University of Automotive Technology, Shiyan, China

**Keywords:** Back Propagation neural network algorithm, network model, green supply chain, intelligent logistics robot, artificial intelligence

## Abstract

With the emergence and development of the Back Propagation neural network (BPNN), its unique learning, generalization, and non-linear characteristics have been gradually excavated and fully applied in the field of prediction. To improve the economic and green benefits of enterprises, the BPNN algorithm is applied to the green supply chain assisted by intelligent logistics robots. The BPNN algorithm can be used to output the characteristics of different information and optimize the green supply chain according to the input parameters and the influencing factors in the network. Firstly, an evaluation index system is established for selecting suppliers, which includes 4 first-level indicators: operational indicators, economic indicators, green indicators, social indicators, and 14 corresponding secondary indicators. Secondly, the evaluation indicator system is modeled through the BPNN. Finally, using the BPNN model, a supply chain enterprise's selection of cooperative enterprises in Xi'an is taken as the research object and simulation. Finally, the output results of the five alternative enterprises are 0.77, 0.75, 0.68, 0.72, and 0.65, respectively. The enterprise with the highest output results is selected as the cooperative enterprise and the enterprise with the second highest output results as an alternate. The green supply chain model based on the proposed BPNN is scientific and effective through specific simulation experiments. It has certain reference significance for the relevant issues related to subsequent optimization of the green supply chain.

## Introduction

With the rapid development of technology and the continuous progress of society, as an important driving force for a new round of scientific and technological revolution and industrial transformation, artificial intelligence (AI) has become an extremely important technological content at present. Under the background of made in China 2025 and the continuous transformation and upgrading of technology in the manufacturing field, the production level and the application efficiency of China's manufacturing have been greatly improved. The widespread use of AI technology has not only brought revolutionary changes to traditional manufacturing, but also affected the transition of modern manufacturing to a smarter, more modular trend (Brito et al., 2020). With the development of AI technology, the robot industry has also developed from programmable robots and sensory robots to intelligent robots, which has become a trend of development at present and in the future (Staal et al., 2020). With the application of intelligent robots in the logistics industry, the operation efficiency of the logistics system has been greatly advanced. Intelligent logistics robots have gradually demonstrated their advantages of speed and convenience in logistics, warehousing, transportation and other fields. While technology and the economy are developing rapidly, “green development” has also become a hot topic in society, and all walks of life are transforming to green.

Ahmet and Alk (2020) believed that supply chain management (SCM) was an important part of reducing costs and increasing profits for most companies in a competitive enterprises environment, and the success of enterprises is directly related to the performance of the supply chain. The introduction of the green SCM theory also provides a new way of thinking for the logistics industry to realize green transformation, so that enterprises can choose the right partners and jointly complete the green transformation. While ensuring the economic benefits of enterprises, they also harvest green benefits. Shao et al. (2019) considered that the idea of the green supply chain could provide a decision-making basis for the bidding and procurement process, and proposed that the green supply chain mainly included three levels: external environment, corporate strategy, and inventory level (Shao et al., 2019). At present, the domestic green supply chain is still in its infancy, and there are fewer studies on the optimization of the existing green supply chain. With the emergence and development of the Back Propagation neural network (BPNN), its unique learning, generalization, and non-linear characteristics have been gradually excavated and fully applied in the field of prediction. The BPNN algorithm can output the characteristics of differentiated information according to different input parameters and various influencing factors in the network, and optimize the green supply chain of the logistics industry. The innovation of this research is to use the predictability of BPNN to predict the choice of different supply chain enterprises, thus achieving the optimal choice.

To improve the economic and green benefits of enterprises, the BPNN algorithm is applied to the green supply chain. Firstly, an evaluation index system is established for selecting suppliers. Secondly, the system is modeled through BPNN. Finally, the BPNN model is used to simulate the selection of partners by a supply chain enterprise in Xi'an, which verifies that the proposed index system model is scientific and effective.

## The Model of Green Supply Chain Using the BPNN

### Construction of Evaluation Index System of Green Supply Chain

The concept of supply chain first appeared in manufacturing, which refers to the whole process of manufacturing enterprises purchasing raw materials, producing and manufacturing, supplying to distributors, transferring to retailers, and finally to consumers. With the gradual deepening of the research on manufacturing operation mode, the academic community combines supply chain with supply management and expresses it as a relationship between suppliers and manufacturing enterprises. Nowadays, manufacturers, supply enterprises, transportation enterprises, distributors, information flows and consumers are all included in the supply chain. In the whole process of SCM, the green supply chain relies on its advanced scientific concepts and technical means, takes economic benefits, social benefits and environmental protection benefits as management goals, and carries out high-efficiency and low-cost overall control of logistics, fund flow and information flow of the entire supply chain (Gao et al., 2021).

The green supply chain is a closed-loop system with four main features: (1) The main goal of traditional SCM is to improve the profitability of enterprises in the supply chain, and the green supply chain not only aims to improve enterprise efficiency, but also adds two new goals: social and environmental benefits. In practice, the three benefits may be contradictory, and the realization of one benefit improvement will often lead to the decline of the other benefit. Green SCM is to comprehensively consider the three benefits and make the three develop together. (2) Traditional supply chain is the product of enterprise organization, and the main body of the green supply chain is more complex. Government intervention, preferences of consumer groups, etc. will have an impact on it. Due to the complexity of the main body in the supply chain, its behavioral goals are also more diversified, thus promoting the common development of multiple benefits. (3) The life cycle of products in the traditional supply chain includes design, raw material procurement, product manufacturing, sales, logistics and after-sales service, while the green supply chain also includes the recovery of waste materials to form a closed loop to minimize energy consumption and reduce environmental hazards. (4) The main factor of traditional SCM is the relationship between subjects, but the green supply chain also includes technical requirements in addition to the relationship between subjects (Cui et al., 2021).

**Figure 1:**
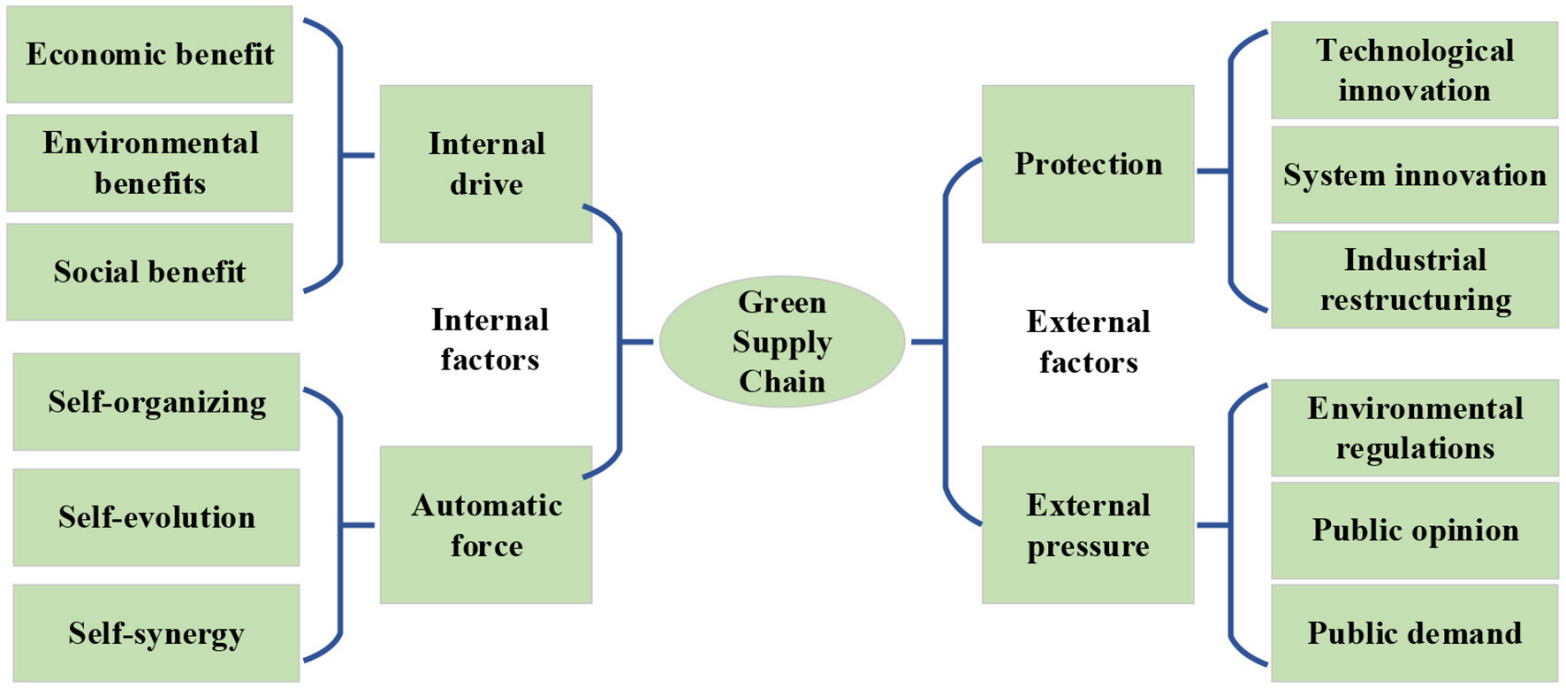
The formation principle of the green supply chain.

The formation of the green supply chain can be divided into internal and external aspects. The specific principle of formation is shown in Figure 1.

In Figure 1, the internal factors that make up the green supply chain include internal driving drive and automatic force. The internal driving force includes economic benefits, social benefits and environmental benefits. The green supply chain improves economic benefits by reducing costs, reducing waste, promoting the market, and improving brand benefits, improving social benefits by improving product security, saving energy and increasing employment, and improving environmental benefits through improving the efficiency of the use of material resources and controlling the entry of highly polluting substances into the supply chain. The automatic forces include self-organization, self-evolved and self-coordinated. The subjects involved in the green supply chain share resources, restrict each other, and also promote each other. When a subject in the supply chain behaves abnormally, the supply chain will adjust itself in time (Fallahpour et al., 2020; Meager et al., 2020).

The external factors that constitute a green supply chain include security force and external pressure. The security force includes technological innovation, institutional innovation, and industrial structure adjustment. Through correct guidance and appropriate incentives, the government promotes the green upgrading of the supply chain, establishes effective environmental regulations, builds standardized incentive mechanisms, guarantees the due benefits of technological innovation of enterprises, makes macro-adjustments to the industrial structure of enterprises, and formulates appropriate industry development plans to achieve the green upgrade of the supply chain (Midya et al., 2021). The external pressure includes environmental regulations and standards, public opinion orientation and public demand. The introduction of relevant regulations and standards can promote the green upgrade of the supply chain, and public opinions on environmental pollution and the use of toxic and hazardous materials can also improve the environmental protection concepts of corporate managers and consumers. With the improvement of public consumption capacity, consumers will also buy products for the green premium of products, and they will be more inclined to choose corporate products with a strong sense of social responsibility, which also ensures the demand for products in the green supply chain (Khan et al., 2021).

**Figure 2:**
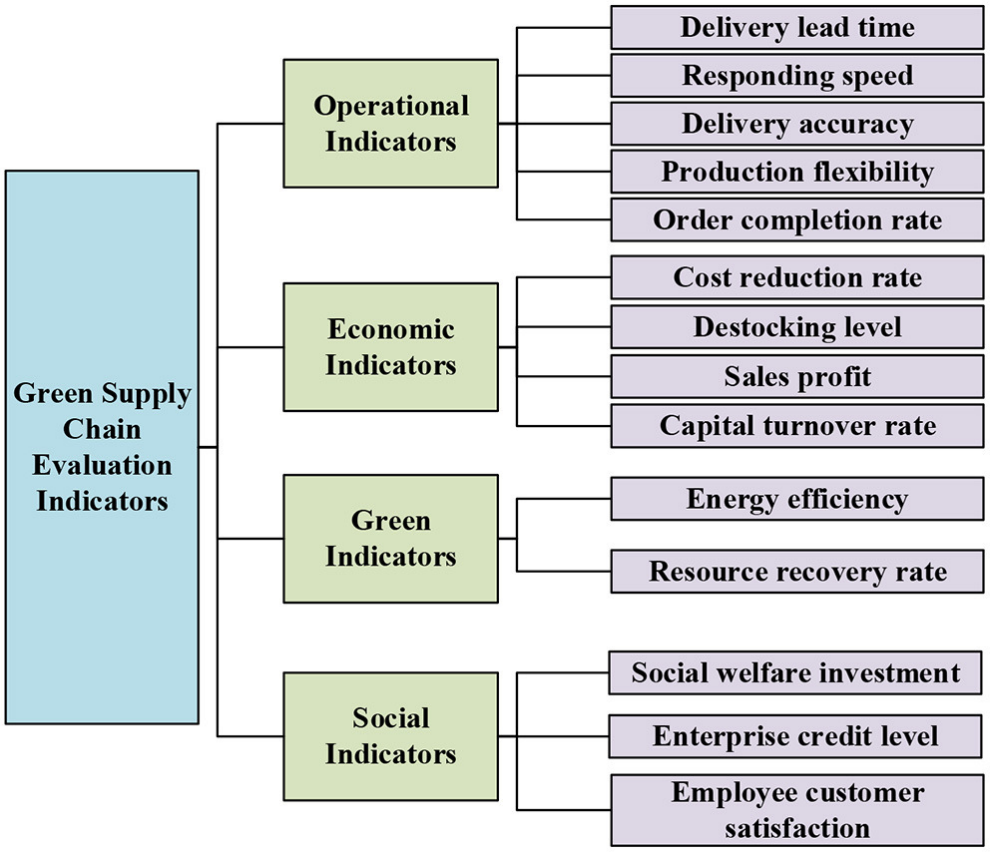
The evaluation indicator system of the green supply chain.

According to the characteristics and formation principle of the green supply chain, the indicator system of the green supply chain is set. The operational indicators, economic indicators, green indicators and social indicators are taken as the first-level indicators of the indicator system of the green supply chain. Among them, operational indicators include five secondary indicators, namely delivery advance, response speed, delivery accuracy, production flexibility and order completion rate. Economic indicators include four secondary indicators, and they are cost reduction rate, destocking level, sales profit margin, and capital turnover rate, respectively. Green indicators include two secondary indicators, namely energy utilization rate and waste resource recovery rate, respectively. Social indicators include three secondary indicators, namely social welfare investment, enterprise reputation level, and employee and customer satisfaction. The specific content is shown in Figure 2.

In Figure 2, the first-level indicators of the evaluation indicator system of the green supply chain are four categories: operational indicators, economic indicators, environmental indicators and social indicators, and the corresponding secondary indicators are a total of 14 categories. Each indicator is independent and does not overlap with each other, but it affects, restricts and interacts with each other. By applying these indicators to BPNN, a green supply chain evaluation model based on BPNN can be constructed.

### The BPNN Model

**Figure 3:**
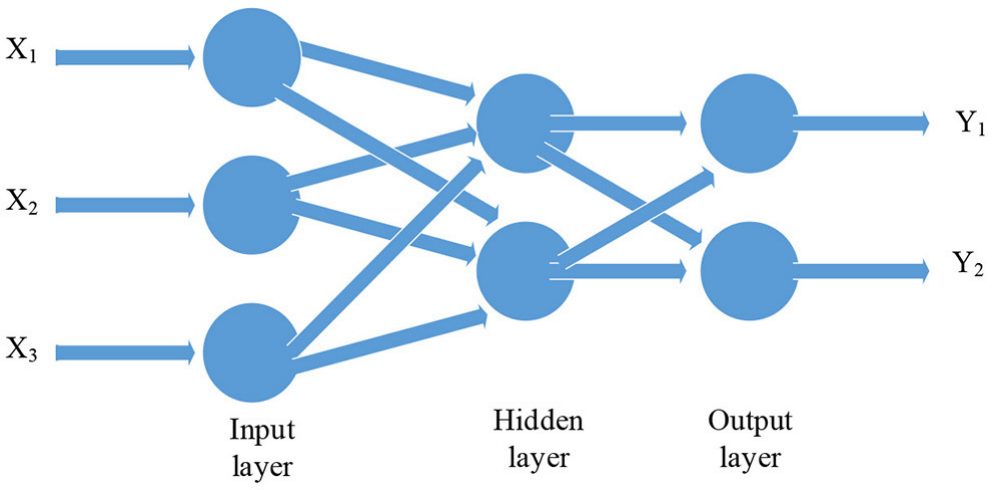
The structure of the BPNN.

For building a green supply chain evaluation model based on BPNN, it is necessary to understand the meaning of BPNN. The concept of the BPNN was first proposed by a group of scientists led by Rumelhart and McClelland in 1986, and it was widely used. The BPNN includes the input layer, the output layer, and the hidden layer. Neurons in the same layer are not connected to each other, and only neighboring upper and lower neurons can connect to each other (Wu et al., 2019). The BPNN can generate different output information by inputting different input data to meet all training sets as much as possible (Tang and Yu, 2021). The structure is shown in Figure 3.

In the BPNN, the hidden layer may be only one layer or multiple layers, each layer may have one or more neurons. But the theory has proved that the BPNN with only one layer of the hidden layer can approximate arbitrary non-linear continuous functions. So in most cases, the BPNN with only one layer of the hidden layer is used. In the input layer, the number of neurons is the same as the number of input parameters, and the number of neurons in the output layer is the same as the number of output parameters (Zhou et al., 2019, 2020).

Generally speaking, the excitation function between the hidden layer and the output layer is the Sigmoid function, which includes two forms, namely the Log-Sigmoid function and the Tan-Sigmoid function. The expression of the Log-Sigmoid function is shown in Equation (1):


(1) $$\begin{array}{r} f\left(x\right)=\frac{1}{1+e^{-x}} \end{array}$$

The expression of the Tan-Sigmoid function is shown in Equation (2):


(2) $$\begin{array}{r} f\left(x\right)=\frac{1-e^{-x}}{1+e^{-x}} \end{array}$$

As the most frequently applied excitation function in the BPNN, the Sigmoid function “extrudes” the input of the upper function and outputs it into a range, thereby completing arbitrary non-linear mapping from input to output (Sakaki et al., 2019).

Neural networks (NNs) also have the function of autonomous learning, and their learning methods can be divided into supervised learning and unsupervised learning. Supervised learning can make the output value of each group of NNs as close as possible to the actual output value. Because the BPNN trains the NN with the input and output of the training set, the number of hidden layers, learning rate, threshold and weights of neurons can be adjusted. Unsupervised learning can only adjust the weights between neurons, and there is no actual output value, so it is impossible to compare the output of the NN with the actual output, so it is impossible to adjust the parameters.

When using the model of the BPNN, there are the following steps: first, the output information is calculated according to the input information, then the threshold and weight are reversely updated between the two adjacent levels according to the error between the actual output and the expected output. When learning, the standard BPNN algorithm uses the descent method of the error function. When the sum of squared errors between the actual output and the expected output is minimized, the learning process ends (Peng et al., 2018; Hirschfeld et al., 2020; Carvalho and Plastino, 2021).

Because the BPNN with only one layer of the hidden layer can approximate arbitrary non-linear continuous functions, resulting in most NN models that only use one layer of the hidden layer, so this time NN models with only one layer of the hidden are also used as examples. a is the number of input layer neurons, b is the number of output layer neurons, and c is the number of hidden layer neurons. Y_mn_ is the weight of the mth input layer neurons to the nth hidden layer neurons. Q_sn_ is the weight of the s-th hidden layer neurons to the nth output layer neurons. x_m1_ to x_ma_ is an input parameter of mth group data. y_m1_ to y_mb_ is the b output parameter of mth group data. Y_n0_ is the threshold of the nth hidden layer neuron. Q_s0_ is the threshold of the sth output neuron. The application process of the BPNN model is shown in the equation.

Step 1: The number of neurons is determined. The number of neurons in the input layer is the same as the number of input values. The number of neurons in the output layer is the same as the number of output parameters. The number of hidden layer neurons is usually estimated by the experience formula, and then the number is determined by temptation. Two experience formulas are shown in Equations (3) and (4):


(3) $$\begin{array}{r} p=\sqrt{a+b}+\varepsilon \;\left(0\leq \varepsilon \leq 10\right) \end{array}$$

(4) $$\begin{array}{r} p=\log_{2}a \end{array}$$

Because the secondary index of the chain evaluation index of this green supply is 14, the number of neurons in the input layer is 14, the number of neurons in the output layer is 1, and the value is 0–10 in turn for ε. The speed of convergence of the NN processing data is tested, and the number of hidden layers is 8. The model of the BPNN is finally set to 14^*^8^*^1.

Step 2: Data pre-processing. Firstly, the input and output of the BPNN are determined, secondly, the corresponding training set is built, and finally, the processing of the data is normalized. Generally, the data is normalized into intervals [0,1] or intervals [−1,1], as shown in Equation (5) to (7):


(5) $$\begin{array}{r} {\text{x}}_{\text{new}}=\frac{\text{x}-{\text{x}}_{\min}}{x_{\max}-{\text{x}}_{\min}} \end{array}$$

(6) $$\begin{array}{r} {\text{x}}_{\text{mid}}=\frac{{\text{x}}_{\max}-{\text{x}}_{\min}}{2} \end{array}$$

(7) $$\begin{array}{r} {\text{x}}_{\text{new}}=\frac{\text{x}-{\text{x}}_{\text{mid}}}{0.5\left({\text{x}}_{\max}-{\text{x}}_{\min}\right)} \end{array}$$

Equation (5) is to normalize the data to [0,1], Equations (6) and (7) are to normalize the data to [−1,1].

Step 3: Parameters of the input network model. Input weight Y_mn_, weight Q_sn_, learning rate η, thresholds, incentive function, maximum recursive times and setting target error.

Step 4: Calculate the output of the hidden layer. First, the weighted sum of the data is calculated by the input layer in the NN, and then the excitation function is used to “compress it” and finally, the output value of the hidden layer is obtained, as shown in Equation (8):


(8) $$\begin{array}{c} {\text{K}}_{\text{m}}=\text{f}({\sum^{\text{​}}}_{\text{k}\;=\;1}^{\text{a}}{\text{Y}}_{\text{km}}{\text{x}}_{\text{k}}+{\text{Y}}_{\text{m0}}) \end{array}$$

In the equation, *K*_m_ is the output value of the mth neuron of the hidden layer. f() is the excitation function, as shown in Equations (1) or (2).

Step 5: Calculate the output value of the output layer neurons, as shown in Equation (9):


(9) $${\text{y}}_{\text{i}}=\text{f}({\sum^{\text{​}}}_{\text{k}\;=\;1}^{\text{n}}{\text{K}}_{\text{k}}{\text{Q}}_{\text{km}}+{\text{Q}}_{m0})$$

Step 6: Calculation error. The output error is calculated according to the output value of the BPNN model and the actual output value. The sum of squares of errors is shown in Equation (10):


(10) $$\text{E}={\sum^{\text{​}}}_{\text{m}\;=\;0}^{{\text{N}}_{\text{t}}}\;{\sum^{\text{​}}}_{\text{n}\;=\;1}^{b}{\left[{\text{t}}_{\text{n}}^{\left(\text{m}\right)}-{\text{y}}_{\text{n}}^{\left(\text{m}\right)}\right]}^{2}$$

In Equation (10), E represents the sum of squares of errors. *N*_*t*_ indicates the total number of training samples. m shows the mth training sample. n is the nth output neuron, and b means the number of output neurons. $y_{n}^{\left(m\right)}$ is the nth network model output of the mth training sample and $t_{n}^{\left(m\right)}$ is the nth actual output of the mth training sample.

Step 7: Distinguish whether the training is over. Distinguish whether the BPNN model training is over or not by the following situations:

(1) Given error maximum > error E.
(2) The number of recursions > the set parameter values.
(3) When the training error E is basically unchanged for many consecutive times, it has converged to the minimum value.

If one of the above conditions is met, skip the next step, otherwise, go to the next step.

Step 8: First, adjust the weights and thresholds in the NN, and then jump back to Step 4. The BPNN algorithm is used to learn according to the gradient descent method of error functions. The partial derivatives of E relative thresholds and weights should be calculated first, as shown in Equations (11) to (14):


(11) $$\begin{array}{ccc} \frac{\partial \text{E}}{\partial {\text{Q}}_{\text{sn}}} & = & {\sum^{\text{​}}}_{\text{p }=\;1}^{N_{t}}[{\text{y}}_{\text{s}}^{\left(\text{p}\right)}-{\text{t}}_{\text{s}}^{\left(\text{p}\right)}]\text{φ}({\sum^{\text{​}}}_{\text{m}=1}^{\text{a}}{\text{Y}}_{\text{nm}}{\text{x}}_{\text{m}}+{\text{Y}}_{\text{n0}}) \end{array}$$

(12) $$\begin{array}{rcl} \frac{\text{∂E}}{\text{∂}{\text{Q}}_{\text{s}0}} & = & \sum_{\text{p }=\;1}^{{\text{N}}_{\text{t}}}\left[{\text{y}}_{\text{s}}^{\left(\text{p}\right)}-{\text{t}}_{\text{s}}^{\left(\text{p}\right)}\right] \end{array}$$

(13) $$\begin{array}{l} \begin{array}{ccc} \frac{\partial \text{E}}{\partial Y_{\text{nm}}} & = & {\sum^{\text{​}}}_{\text{p}\;=\;1}^{{\text{N}}_{\text{t}}}{\sum^{\text{​}}}_{\text{s}\;=\;1}^{\text{b}}[{\text{y}}_{\text{s}}^{\left(\text{p}\right)}-{\text{t}}_{\text{s}}^{\left(\text{p}\right)}]\text{φ}({\sum^{\text{​}}}_{\text{m}=1}^{\text{a}}{\text{Y}}_{\text{nm}}{\text{x}}_{\text{m}}+{\text{Y}}_{\text{n}0}) \end{array}\\ \;\begin{array}{c} {}[1-\varphi ({\sum^{\text{​}}}_{\text{m }=\;1}^{\text{a}}{\text{Y}}_{\text{nm}}{\text{x}}_{\text{m}}+{\text{Y}}_{\text{n}0})]x_{m} \end{array} \end{array}$$

(14) $$\begin{array}{l} \begin{array}{ccc} \frac{\partial \text{E}}{\partial {\text{Y}}_{\text{n}0}} & = & {\sum^{\text{​}}}_{\text{p}=1}^{{\text{N}}_{\text{t}}}{\sum^{\text{​}}}_{s\;=\;1}^{\text{b}}[y_{\text{s}}^{\left(\text{p}\right)}-t_{\text{s}}^{\left(\text{p}\right)}]\text{φ}({\sum^{\text{​}}}_{\text{m}\;=\;1}^{a}{\text{Y}}_{\text{nm}}{\text{x}}_{\text{m}}+{\text{Y}}_{\text{n}0}) \end{array}\\ \;\begin{array}{c} \left[1-\varphi ({\sum^{\text{​}}}_{\text{m}=1}^{\text{a}}{\text{Y}}_{\text{nm}}{\text{x}}_{\text{m}}+{\text{Y}}_{\text{n}0})\right] \end{array} \end{array}$$

In the equations, E represents the sum of squares of errors. *N*_*t*_ indicates the total number of training samples. m shows the mth training sample. n is the nth output neuron, and b means the number of output neurons. The thresholds *Y*_*n*0_, *Q*_*s*0_ and weights *Y*_*nm*_, *Q*_*sn*_ are modified, as shown in Equations (15) to (18):


(15) $$\begin{array}{r} {\text{Q}}_{\text{sn}}^{\text{new}}={\text{Q}}_{\text{sn}}^{\text{old}}-\eta \frac{\text{∂E}}{\text{∂}{\text{Q}}_{\text{sn}}} \end{array}$$

(16) $$\begin{array}{r} {\text{Q}}_{\text{s}0}^{\text{new }}={\text{Q}}_{\text{s}0}^{\text{old }}-\eta \frac{\text{∂E}}{\text{∂}{\text{Q}}_{\text{s}0}} \end{array}$$

(17) $$\begin{array}{r} {\text{Y}}_{\text{nm}}^{\text{new}}={\text{Y}}_{\text{nm}}^{\text{old}}-\eta \frac{\text{∂E}}{\text{∂}{\text{Y}}_{\text{nm}}} \end{array}$$

(18) $$\begin{array}{r} {\text{Y}}_{\text{n}0}^{\text{new }}={\text{Y}}_{\text{n}0}^{\text{old }}-\eta \frac{\text{∂E}}{\text{∂}{\text{Y}}_{\text{n}0}} \end{array}$$

Step 9: Verify the feasibility of the algorithm. The BPNN model that has been trained is used to calculate the output according to the input of the training set, and then the output value of the NN model with the actual output value are compared and calculated. Finally, the error is calculated and the rationality of its structure is analyzed.

**Figure 4:**
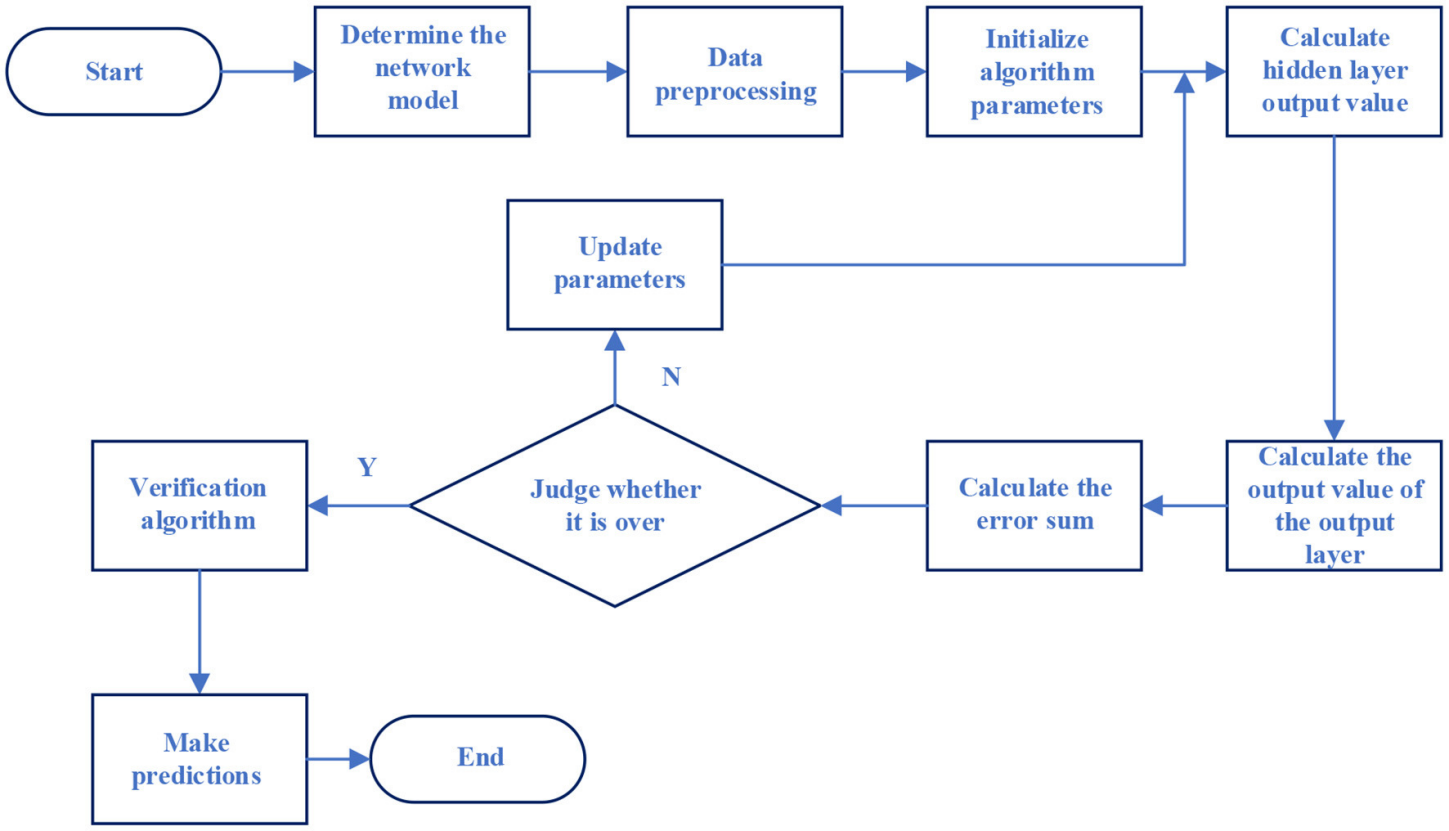
The operation flow of the BPNN model.

Step 10: After the algorithm training is over, the completed NN model is used to solve the problem. The operation flow of the BPNN model is shown in Figure 4.

The reason why the BPNN model can be used so widely is due to its following characteristics:

(1) Good non-linear mapping ability. In the BPNN model, the threshold of each neuron, the weights between neurons, etc., can be stored as specific information.
(2) Distributed storage information. In the BP neural network model, the threshold of each neuron, the weights between neurons, etc. can be expressed as stored as specific information.
(3) Autonomous learning ability. The BPNN model will calculate the output according to the input of the training set, and then compare it with the actual output to reversely update the threshold and weights in the model until the network model gradually stabilizes.
(4) Synchronization. All neurons in the BPNN can receive and process information separately, so neurons in the same layer can calculate the received data synchronously, and then transmit the calculated results to the lower neurons together.
(5) Good fault tolerance. Even if the BPNN model is partially damaged or the information received is lost, it can work without much impact (Huang et al., 2020; Sze et al., 2020; Samek et al., 2021).

**Figure 5:**
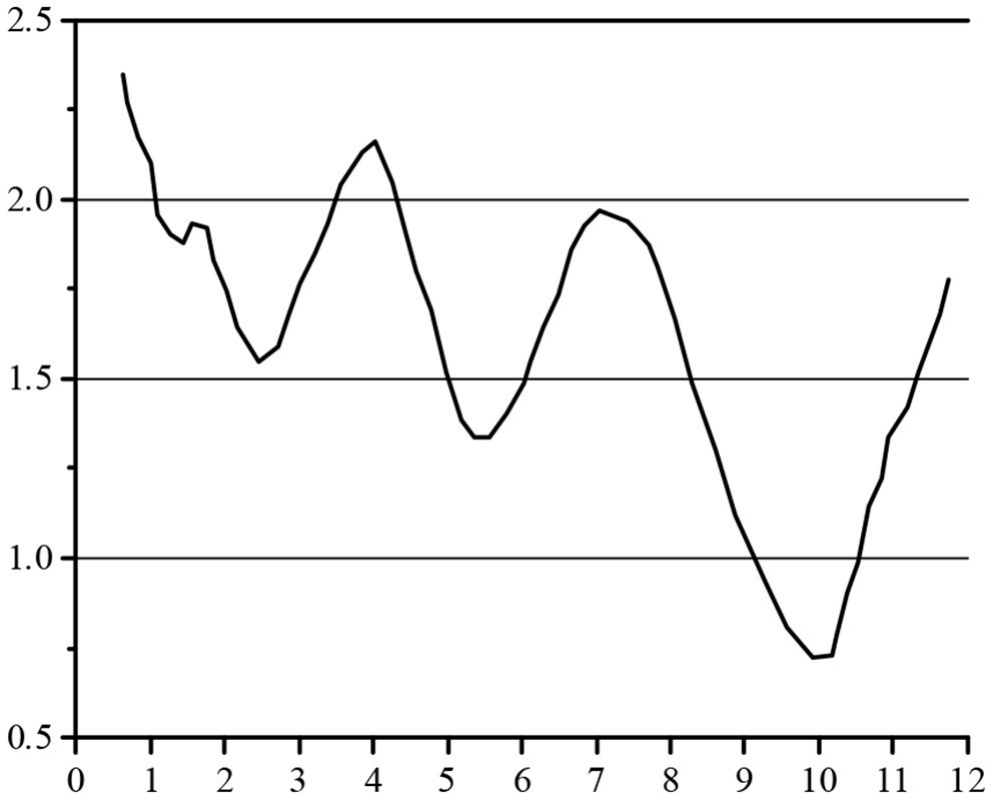
The function curve of the sum of errors.

The BPNN has the above advantages and solves many problems. However, with people's continuous research on it, it is found that there are many shortcomings a. It is mainly reflected in the following aspects:

(1) The convergence speed of the sum of errors is very slow during training. To maintain stability, the learning rate is a small fixed value, which leads to the convergence speed of the sum of errors is very slow during BPNN training.
(2) The sum of errors can easily enter the local minimum value. The function diagram of the sum of errors is a continuous but uneven curve, which contains N local minimal values and an overall minimal value. The purpose of model training is to find the overall minimal value of the sum of errors. In the process of training, it starts from a point in the function curve of the sum of errors, and moves to a minimum value in the descending direction. This minimum value may be the overall minimum value or a local minimum value, so the sum of errors is easy to enter the local minimum value in the process of training (Alarifi et al., 2020). The details are shown in Figure 5.
(3) The training time of the BPNN is long. Because the number of layers of the hidden layer and the number of neurons are not fixed, even if the number of layers is generally only used one layer, however, the experience formulas should still be used to calculate the valuation to determine the number of neurons in the hidden layer, and then train them many times to test the most suitable number of neurons. Therefore, the training time of the BPNN is long.
(4) The NN model is unstable. When the BPNN model gradually stabilizes through training, and then inputs new training data, the previous threshold and weights cannot be applied, and the NN model cannot be applied, so the new training data has to be combined with the previous training data for retraining. Therefore, the BPNN model is unstable (Zielonka et al., 2020).
(5) There is an endless cycle in the NN. When the Sigmoid function is used as an incentive function, if the learning rate or weight is too large, the derivative of the Sigmoid function will tend to be 0, and the correction values of the threshold and weights will also tend to be 0. At this time, the NN will fall into an endless cycle.

In view of the above situation, the adaptive adjustment method of the learning rate and the additional momentum method are generally used to improve it. The additional momentum method is used to adjust the weights and thresholds, as shown in Equations (19) to (22):


(19) $$\begin{array}{r} \text{Δ}{\text{Q}}_{\text{sn}}^{\text{new }}=\text{αΔ}{\text{Q}}_{\text{sn}}^{\text{old }}-\eta \frac{\text{∂E}}{\text{∂}{\text{Q}}_{\text{sn}}} \end{array}$$

(20) $$\begin{array}{r} \text{Δ}{\text{Q}}_{\text{s}0}^{\text{new}}=\text{αΔ}{\text{Q}}_{\text{s}0}^{\text{old}}-\eta \frac{\text{∂E}}{\text{∂}{\text{Q}}_{\text{s}0}} \end{array}$$

(21) $$\begin{array}{r} \text{Δ}{\text{Y}}_{\text{nm}}^{\text{new}}=\text{αΔ}{\text{Y}}_{\text{nm}}^{\text{old }}-\eta \frac{\text{∂E}}{\text{∂}{\text{Y}}_{\text{nm}}} \end{array}$$

(22) $$\begin{array}{r} \text{Δ}{\text{Y}}_{\text{n}0}^{\text{new}}=\text{αΔ}{\text{Y}}_{\text{n}0}^{\text{old }}-\eta \frac{\text{∂E}}{\text{∂}{\text{Y}}_{\text{n}0}} \end{array}$$

α is a momentum factor with a value (0,1); η is a learning rate with a value (0,1); and E represents the sum of squares of the training error. Due to the influence of momentum factors, when the training drops to a local minimal value, the minimal value is still searched until the overall minimal value is reached (Huang et al., 2019). η will affect the training speed and model effect. The automatic adjustment method of the learning rate can deal with this problem:


(23) $$\eta_{k+1}=\{\begin{array}{c} 1.04\eta_{k},E_{k+1}<E_{k}\\ 0.8\eta_{k},E_{k+1}>1.05E_{k}\\ \eta ,\text{ other} \end{array}$$

k shows the kth algorithm recursive. When the sum of squares of the training error is less than the sum of the recursive squares of the previous algorithm, the learning rate can be appropriately improved. When it is >1.05 times the sum of the recursive error squares of the previous algorithm, the learning rate should be appropriately reduced. In other cases, the learning rate should remain unchanged.

## Verification of Experimental Data and Simulation of Model

### Verification of Experimental Data

A supply chain enterprise in Xi'an as an example is taken to select suitable supply chain partners for it. The alternative collaborators are A-E. According to the existing indicator system, the BPNN model is established. The number of neurons in the input layer is 14, the number of neurons in the hidden layer is 8, and the number of neurons in the output layer is 1. The final output value of the model is the final evaluation result, and the priority is given to the high score.

The data are collected in the form of questionnaires. The questionnaire adopts the Likert five-point scale. Each question ranges from totally disagree to totally agree, representing 1-5 points, respectively. The specific content of the questionnaire is shown in Table 1.

**Table 1:** Questionnaire of self-evaluation indicators of the green supply chain.

**No**.	**Problem description**	**Totally disagree**	**Broadly disagree**	**Largely agree**	**Broadly agree**	**Totally agree**
1	Customers are very satisfied with the quality of the product					
2	Customers are very satisfied with the price of the product					
3	Customers are very satisfied with the timeliness of delivery					
4	Customers are very satisfied with the accuracy of delivery					
5	The needs of customers are satisfied for different batches and different product combinations					
6	The company always pays attention to the market and responds quickly					
7	The company can successfully solve the temporary increase in orders					
8	The company can meet orders of different batches at any time					
9	The company keeps inventory to a minimum while maintaining customer demand					
10	The company's transportation costs are reduced to a minimum					
11	The company minimizes loss of the product					
12	The company can make accurate forecasts of inventory levels					
13	There is no problem with the turnover of the company's funds					
14	The company's control of sales profits is very strict					
15	The company can rationally use equipment and tools to improve efficiency					
16	The company saves or recycles recyclable resources					
17	The benefits within the company are very good					
18	Employees are very satisfied with the company's salary and benefits					
19	The company has a certain reputation in the society					
20	The company and its partners can achieve mutual benefit and win-win results					

**Figure 6:**
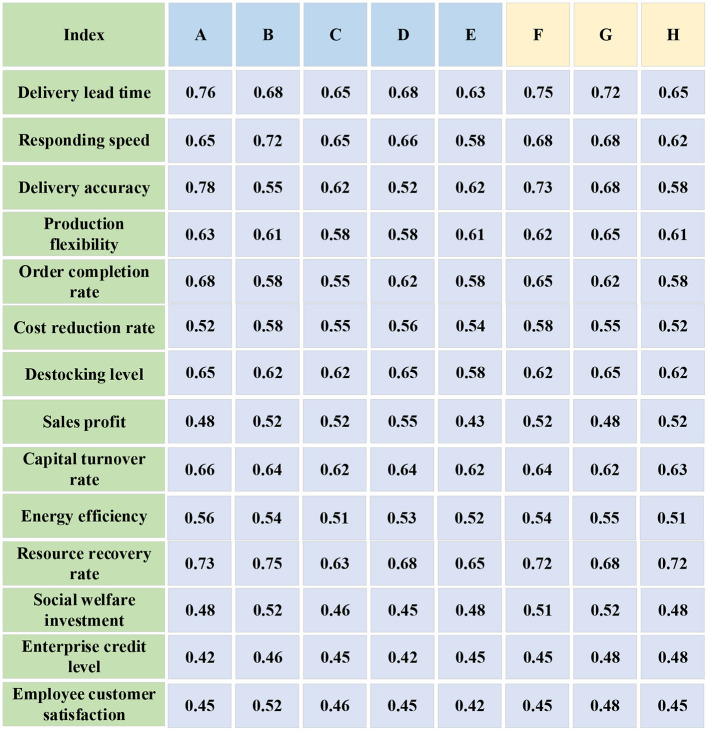
The data of testing and training sample.

In Table 1, the problems contain the actual problems shown by all 14 secondary indicators. The questionnaire is distributed to the management of the alternative partner, 20 copies per enterprise, and finally, the sample data of the test partner is obtained by sorting out the statistics of the questionnaire. In addition, F, G and H enterprises in the same industry are selected to investigate as training samples. The test sample data and training sample data are normalized after they are obtained. The final data is shown in Figure 6.

**Figure 7:**
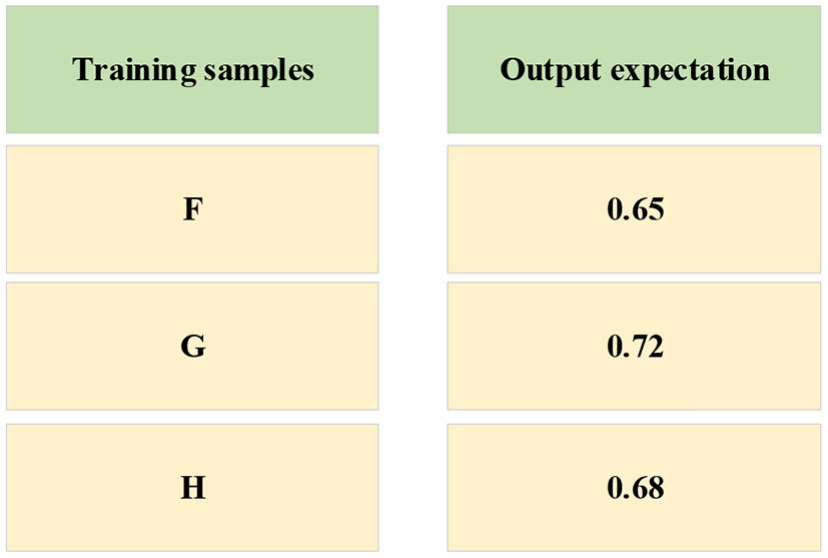
The output value of training sample.

The training data is input into the NN and the expected output is shown in Figure 7.

### Simulation and Analysis of Model

**Figure 8:**
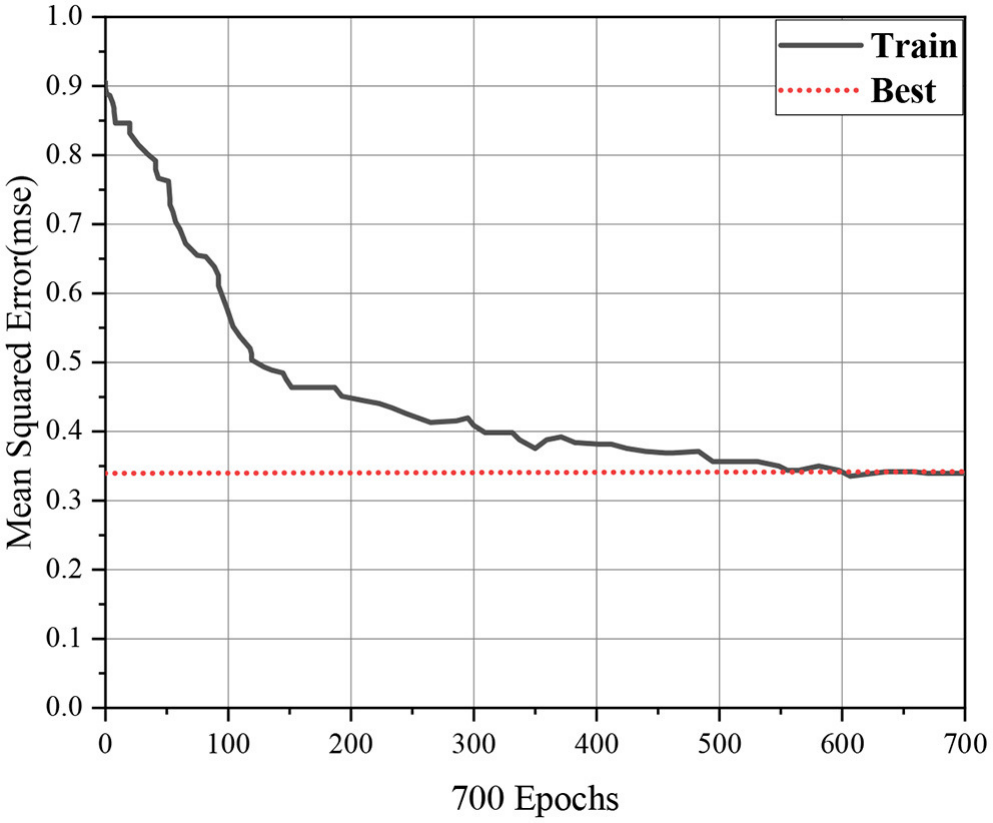
The convergence diagram of the target error of the BPNN.

In Figure 5, the data of the three enterprises F, G, and H are brought into the NN as the input value, and the output value in Figure 6 is taken as the target. When inputting the training function, the target error is 0.01. After 700 times of training, the requirements of the error are met, and the training is completed. The convergence diagram of the target error of the BPNN is shown in Figure 8.

**Figure 9:**
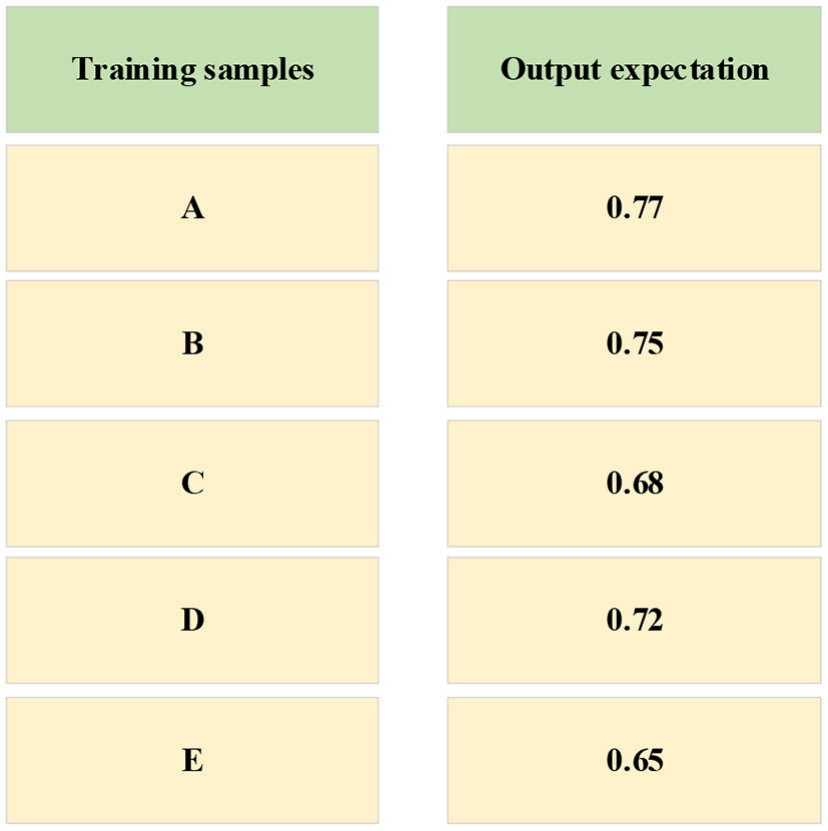
Results of the output.

The test data of the five enterprises A-E are brought into the trained BPNN model, and the expected value is obtained as Figure 9.

Figure 9 indicates that enterprise A has the highest evaluation, with a score of 0.77, which can be used as a supply chain partner, and enterprise B ranks second in the evaluation, with a score of 0.75, which can be used as an alternative enterprise for supply chain partners. The evaluations of C, D and E enterprises are 0.68, 0.72 and 0.65, respectively. The evaluation score is lower than that of company A and company B, so it can be given up in this selection of the supply chain partner. Finally, this experiment selected the optimal cooperative enterprise for the supply chain through the BPNN.

BPNN has strong self-learning and self-improvement capabilities. In other words, the quality and quantity of training samples brought in during the BPNN training process largely determine the output quality of the evaluation model. Therefore, to make enterprises choose more suitable supply chain partners, enterprises should use the evaluation in the process of supplier selection and the evaluation in the whole process of contract performance to establish a complete supplier evaluation database. Meanwhile, the BPNN is continuously trained as a sample to improve the supplier's BPNN selection model. And enterprises should also do a good job in the training of staff, carry out special training courses, professional lectures, experience sharing sessions, etc., to improve staff's understanding of the BPNN system and practical skills. On the basis of systematic training for the existing personnel, the introduction of high-level talents should also be carried out in a targeted manner, and the talent structure should be adjusted.

## Conclusion

The BPNN algorithm is used to output various information features and optimize the green supply chain according to the input parameters and the different influencing factors in the network. Firstly, by combining the management practice of supply chain enterprises, an evaluation index system of the green supply chain is established, and then the system is combined with the BPNN algorithm to build a BPNN model. Secondly, a supply chain enterprise in Xi'an as an example is taken to select suitable supply chain partners for it, the relevant data of the five alternative enterprises is input into the trained NN. Finally, the output results are 0.77, 0.75, 0.68, 0.72, and 0.65, respectively. The enterprise with the highest output results is selected as the cooperative enterprise. Through specific experiments, the scientific and effectiveness have been proved. Due to some limitations, the involved evaluation indicators are not comprehensive enough. In the future, the scope of research will be expanded, the evaluation indicators of the green supply chain will be added, and the number of influencing factors and neurons will be added. It has certain reference significance for the relevant issues related to subsequent optimization of the green supply chain.

## Data Availability Statement

The raw data supporting the conclusions of this article will be made available by the authors, without undue reservation.

## Author Contributions

The author confirms being the sole contributor of this work and has approved it for publication.

## Conflict of Interest

The author declares that the research was conducted in the absence of any commercial or financial relationships that could be construed as a potential conflict of interest.

## Publisher's Note

All claims expressed in this article are solely those of the authors and do not necessarily represent those of their affiliated organizations, or those of the publisher, the editors and the reviewers. Any product that may be evaluated in this article, or claim that may be made by its manufacturer, is not guaranteed or endorsed by the publisher.
